# Validation of Viral Inactivation Protocols for Therapeutic Blood Products against Severe Acute Respiratory Syndrome Coronavirus-2 (SARS-CoV-2)

**DOI:** 10.3390/v14112419

**Published:** 2022-10-31

**Authors:** Wendimi Fatimata Belem, Ching-Hsuan Liu, Yee-Tung Hu, Thierry Burnouf, Liang-Tzung Lin

**Affiliations:** 1 International PhD Program in Biomedical Engineering, College of Biomedical Engineering, Taipei Medical University, Taipei 110, Taiwan; 2 Department of Microbiology and Immunology, School of Medicine, College of Medicine, Taipei Medical University, Taipei 110, Taiwan; 3 Graduate Institute of Medical Sciences, College of Medicine, Taipei Medical University, Taipei 110, Taiwan; 4 Graduate Institute of Biomedical Materials and Tissue Engineering, College of Biomedical Engineering, Taipei Medical University, Taipei 110, Taiwan

**Keywords:** COVID-19 convalescent plasma/serum, neutralizing antibodies, pathogen reduction technology, therapeutic blood product

## Abstract

Therapeutic blood products including convalescent plasma/serum and immunoglobulins concentrated from convalescent plasma, such as intravenous immunoglobulins or hyperimmune globulins, and monoclonal antibodies are passive immunotherapy options for novel coronavirus disease 2019 (COVID-19). They have been shown to improve the clinical status and biological and radiological parameters in some groups of COVID-19 patients. However, blood products are still potential sources of virus transmission in recipients. The use of pathogen reduction technology (PRT) should increase the safety of the products. The purpose of this study was to determine the impact of solvent/detergents (S/D) procedures on SARS-CoV-2 infectivity elimination in the plasma of donors but also on COVID-19 convalescent serum (CCS) capacity to neutralize SARS-CoV-2 infectivity. In this investigation, S/D treatment for all experiments was performed at a shortened process time (30 min). We first evaluated the impact of S/D treatments (1% TnBP/1% TritonX-45 and 1% TnBP/1% TritonX-100) on the inactivation of SARS-CoV-2 pseudoparticles (SARS-CoV-2pp)-spiked human plasma followed by S/D agent removal using a Sep-Pak Plus C18 cartridge. Both treatments were able to completely inactivate SARS-CoV-2pp infectivity to an undetectable level. Moreover, the neutralizing activity of CCS against SARS-CoV-2pp was preserved after S/D treatments. Our data suggested that viral inactivation methods using such S/D treatments could be useful in the implementation of viral inactivation/elimination processes of therapeutic blood products against SARS-CoV-2.

## 1. Introduction

SARS-CoV-2 is a pathogenic virus responsible for COVID-19 [1]. The disease has become a serious public health concern on all continents, causing more than 6.5 million deaths as of 2022 [2]. The causative agent of COVID-19 belongs to the enveloped virus, single-stranded, and positive (+) sense RNA, encoding four structural proteins called spike (S), envelope (E), membrane (M), and nucleocapsid (N). All these proteins are essential for producing a structurally complete viral particle [3]. The S protein plays a key role in transmission [3,4]. It binds to the SARS-CoV-2 cell surface receptor, angiotensin-converting enzyme 2 (ACE-2), through the receptor-binding domain (RBD) of the spike protein S1 subunit to mediate viral entry [5]. Many neutralizing antibodies (nAbs) are also directed against the different regions of this protein [6]. Although the RBD protein is the immunodominant protein of SARS-CoV-2, evidence exists for a substantial role of other regions of S-protein in antigenicity [7]. There was a good correlation between the neutralizing effects of COVID-19 convalescent plasma (CCP) and anti-S Ab [8], anti-S1, or anti-RBD IgG contents [9,10,11].

Since the appearance of the disease, several strategies have been implemented against SARS-CoV-2, including therapy through CCP transfusion or infusion of Ig concentrated from CCP [12,13,14]. Like any other therapy, CCP may induce side effects in some patients, but a large number of randomized clinical studies has concluded that CCP exhibits the same safety profile than that of standard plasma for transfusion, including in children [15,16,17,18,19,20]. Therapy based on CCP has shown beneficial effects in COVID-19 disease management for severely [21,22] or critically [22] ill patients. Several clinical studies, but not all [15,20], have demonstrated an effective reduction of viral load [23,24,25], clinical outcome improvement [14], and decreased mortality rate [24,25]. In spite of uncertainties existing on the efficacy of CCP in late-stage COVID-19 disease, there is a growing consensus that passive polyclonal immunotherapy using CCP has emerged as possible and promising treatment for COVID-19 treatment in some patient populations, most specifically when they are administered early in the progression of the disease (before seroconversion) and in immunocompromised patients [13,20,26]. Passive polyclonal immunotherapies may provide therapeutic benefit to patients with preexisting immunosuppression who have weak responses to SARS-CoV-2 vaccines and are at high risk of COVID-19 complications [27]. RCTs have evaluated the efficacy of CCP in hospitalized patients with preexisting immunosuppression [28,29], and data suggest decreased mortality linked to CCP transfusion compared to standard of care or placebo, supporting “CCP transfusion in addition to the usual standard of care for hospitalized patients with pre-existing immunosuppression, as a weak recommendation with moderate certainty of evidence” by the Association for the Advancement of Blood and Biotherapies (AABB) [30]. Recently, the CORIPLASM study [31] identified that in hospitalized COVID-19 patients with underlying immunosuppression, CCP transfusion was associated with reduced mortality compared to the standard of care. CCP may therefore represent a realistic treatment option for immunocompromised patients affected by SARS-CoV-2 and its variants. Several regulatory agencies, including the US FDA, authorize the evaluation of CCP in immunocompromised patients [32].

The administration of convalescent products can certainly exert a potent efficacy due to their contents in specific nAbs against SARS-CoV-2 that are in the form of IgG, IgM, and IgA classes [9,10]. According to the initial US Food and Drugs Administration (FDA) recommendation, nAb titer in therapeutic CCP should be at ≥1:160 [33], although different cutoffs have been suggested later due to manufacturer differences [34].

To our knowledge, there has been no reported case of SARS-COV transmission after CCP transfusion [35], but some studies detected the presence of SARS-CoV-2 genome in CCP [36]. Although the presence of the genome does not mean infectivity, it is preferable anyway to use strategies to increase the safety of CCP against any virus transmissible by transfusion of CCP. The approaches to minimize the risk of residual transfusion of transmissible viruses by plasma therapeutic products while maintaining a high titer of functional Abs against SARS-CoV-2 should be demonstrated to support the validity of the therapy, as they still remain challenges that need to be addressed.

Since the onset of the disease, viral inactivation methods of SARS-CoV-2 have been studied within the context of ensuring safe handling of biological samples (plasma and serum) taken from potentially infected individuals. Among them are treatments with detergents alone [37,38] and solvent/detergents (S/D) [39]. However, these protocols, specifically evaluated for biological samples, do not take into account the specific processing conditions of the licensed S/D methods applied to plasma for transfusion. Most importantly, nothing is also known about the impact of the S/D treatment on the neutralizing activity of Abs in COVID-19 convalescent blood products. Today the impact of different pathogen reduction technology (PRT) using photochemical treatments has been evaluated on SARS-CoV-2 inactivation and maintenance of the immunological properties of CCP, in particular nAbs [40,41]. Moreover, therapy based on convalescent plasma (CP) or hyperimmune globulins (HIG) could be associated with some side effects such as transfusion-related acute lung injury (TRALI), transfusion-associated circulatory overload (TACO), hemolytic transfusion reactions, Ab-dependent enhancement, and allergic reactions [42]. Some of these adverse effects (TRALI, TACO, and allergic reactions) were observed following the CCP transfusion [43]. S/D-treated plasma exhibits major advantages in reducing most of these side effects compared to freshly frozen plasma (FFP) or plasma treated with other PRT [44].

Our research work aims (a) to address the capacity of the S/D treatment to inactivate SARS-CoV-2, and (b) to unveil its impact on the neutralization capacity of COVID-19 convalescent serum (CCS). To achieve this goal and compare the impacts, we employed inactivation protocols using the organic solvent Tri-n-butyl phosphate (TnBP) combined with the detergents (TritonX-45 or Triton X-100), followed by Sep-Pak C18 Plus Light Cartridge filtration to achieve S/D removal as used for industrial applications.

## 2. Materials and Methods

### 2.1. Reagents and Chemicals

Dulbecco’s modified Eagle’s medium (DMEM), fetal bovine serum (FBS), gentamycin, and amphotericin B were obtained from GIBCO-Invitrogen (Carlsbad, CA, USA). Dulbecco’s phosphate-buffered saline (PBS) was obtained from Hyclone (Logan, UT, USA). Cell Counting Kit-8 (CCK-8) and Triton X-45 were purchased from Sigma–Aldrich. Polyethylenimine (PEI) transfection reagent was obtained from Polysciences (Warrington, PA, USA). Tri-n-butyl phosphate (TnBP) and Triton X-100 were from Merck KGaA (Darmstadt, Germany).

### 2.2. Cell Culture

Human hepatoma cells Huh-7 and human embryonic kidney HEK 293FT cells (Invitrogen/Thermo Fisher Scientific) were maintained in DMEM containing 10% FBS, 1% gentamycin, and 1% amphotericin B and incubated at 37 °C in 5% CO_2_ incubator as previously described [45]. HEK 293FT cells were additionally supplemented with 0.5 mg/mL G418 sulfate (InvivoGen; San Diego, CA, USA) [45]. A basal medium of DMEM containing 2% FBS with antibiotics was used in all viral infection assays.

### 2.3. Normal Plasma and Convalescent Serum Preparation

FFP was obtained from the Taipei Blood center (Guandu, Taiwan Blood Services Foundation, Taipei, Taiwan). The plasma was prepared from donations collected from volunteers who fulfilled the donation requirements in the presence of citrate anticoagulant solution. Patient-derived CCS was obtained from the National Health Research Institute Biobank (Miaoli, Taiwan). All samples were stored at −80 °C until the day of the experiment.

### 2.4. Preparation of SARS-CoV-2 Pseudoparticles (SARS-CoV-2pp)

The production of SARS-CoV-2pp was performed using a previously described method [46] with a few modifications. Briefly, HEK 293FT cells were seeded in 10 cm dishes (5 × 10^6^ cells/dish) and co-transfected with 10 μg *Env*-defective human immunodeficiency virus (HIV) proviral construct that encodes firefly luciferase [47] and 10 μg SARS-CoV-2 S plasmid using the PEI transfection reagent. The SARS-CoV-2 S expressor plasmid is generated based on the first isolate Wuhan-Hu-1 (GenBank: MN908947.3) modified with 19aa truncation at its cytoplasmic tail [48] through gene synthesis (Genomics, New Taipei City, Taiwan) and standard cloning techniques. The cells were incubated overnight before the transfection mixture was removed and replaced with DMEM supplemented with 2% FBS. SARS-CoV-2pp in supernatants were collected at 72 h and 96 h post-transfection by PEG-8000 (Sigma) concentration and resuspended in PBS.

Pseudovirus titers were determined by analysis of luciferase activity after infection of Huh-7 cells. Briefly, the serial dilution of SARS-CoV-2pp stock solution was used to infect the monolayer cells (1 × 10^4^ cells/well of a 96-well plate) for 2 h, followed by washing twice with PBS, and was overlaid with basal medium. The plate was incubated for 72 h, and the cells were harvested and assayed for luciferase activity using the Luciferase Assay System (Promega) according to the manufacturer’s instructions. A luciferase reporter signal of at least 1 log_10_ higher than the mock controls was deemed positive for successful SARS-CoV-2pp entry and was then used to determine the 50% tissue culture infectious dose (TCID_50_) based on the Reed–Muench method [24].

### 2.5. S/D Inactivation Protocols of the SARS-CoV-2pp-Spiked FFP

Normal FFP was spiked with SARS-CoV-2pp at a ratio of 9:1 (normal plasma:SARS-CoV-2pp). SARS-CoV-2pp-spiked FFP was treated with S/D agents according to the previously published conditions [49]. Briefly, the samples were mixed with S/D at a final concentration of 1% (*v*/*v*) each: 1% TnBP + 1% TritonX-45 (S/D_45_) or 1% TnBP + 1% Triton X-100 (S/D_100_). The S/D-treated SARS-CoV-2pp-spiked plasma was incubated at 31°C for 30 min before reverse-phase chromatography using a Sep-Pak C18 Plus Light Cartridge, 130 mg Sorbent, 55–105 µm (Waters Corporation, Milford, MA, USA) to stop the reaction and remove S/D.

### 2.6. Cytotoxicity Assay

Cytotoxicity of FFP and S/D treatments (S/D_45_ or S/D_100_) with or without C18 cartridge filtration was tested in Huh-7 cells using the CCK-8 assay. Huh-7 cells (1 × 10^4^ cells/well of a 96-well plate) were treated with 10X diluted experimental samples comprising normal FFP, normal FFP + S/D, or normal FFP + S/D followed by C18 cartridge filtration. The diluted samples were added to the cells. After 72 h of incubation, 10 µL of CCK-8 solution was added to each well. The plate was further incubated for 2 h followed by the measurement of absorbance at 450 nm with a spectrophotometer. Cell viability was normalized to the Mock treatment group.

### 2.7. Infectivity Assay

The filtered S/D-treated SARS-CoV-2pp-spiked FFP was diluted 10X in basal medium for infectivity assay to determine the inactivation ability of S/D treatment. Huh-7 cells (1 × 10^4^ cells/well of a 96-well plate), which are susceptible to SARS-CoV-2 infection [50], were infected with the 10X diluted samples (final multiplicity of infection (MOI) of SARS-CoV-2pp was 0.005) for 1.5 h at 37 °C, before the inoculum was removed, the cells were washed twice with PBS and overlaid with basal medium. Following another 72 h of incubation, the cells were harvested and assayed for luciferase activity as described above. Cells fixed with 4% paraformaldehyde (PFA) before SARS-CoV-2pp infection, thus precluding viral entry, served as a negative control.

### 2.8. Effect of S/D Inactivation Protocols on the Neutralizing Activity of CCS

The impact of S/D treatments (S/D_45_ or S/D_100_) and C18 cartridge filtration on SARS-CoV-2 nAbs was evaluated using patient-derived CCS. CCS was diluted 9X in PBS and treated with S/D and C18 cartridge filtration as described above. The treated CCS was then diluted 10X in basal medium (final dilution 1:90), mixed with SARS-CoV-2pp (final MOI 0.025), and preincubated at 37 °C for 1 h in an Eppendorf tube. The CCS-virus mixture was then used to infected Huh-7 cells (1 × 10^4^ cells/well of a 96-well plate) for 2 h at 37 °C. After the inoculum was removed, the cells were washed twice with PBS and overlaid with basal medium. Following another 72 h of incubation, the cells were harvested and assayed for luciferase activity as described above.

### 2.9. Statistical Analysis

Statistical analysis was performed by one-way ANOVA followed by post hoc tests. Data are presented as mean ± standard deviation (SD). *p* values less than 0.05 were considered statistically significant.

## 3. Results

### 3.1. Production of SARS-CoV-2pp

**Figure 1:**
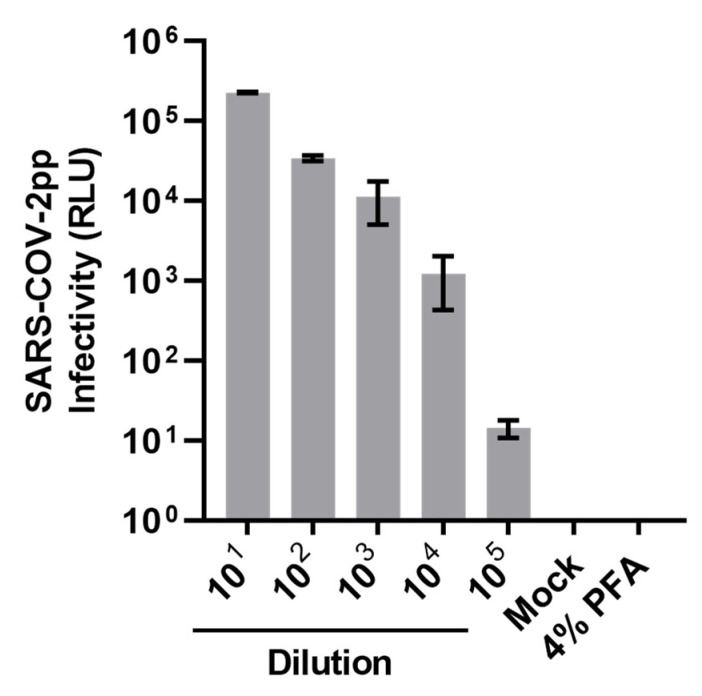
**Titration of SARS-CoV-2 pseudoparticles (SARS-CoV-2pp).** The serial dilution of SARS-CoV-2pp stock was used to infect Huh-7 cells for 2 h. Viral infectivity was measured by luciferase reporter activity (relative light units; RLU) after 72 h incubation. Data shown are collected in triplicate and expressed as mean ± standard deviation (SD). Paraformaldehyde (PFA; 4%) fixed cells infected with 10× diluted SARS-CoV-2pp stock was included as a negative control.

To evaluate the efficacy of S/D treatment plus C18 filtration protocol for inactivating potential SARS-CoV-2 particles in CCP, we produced lentivirus-based SARS-CoV-2pp to spike normal FFP as a surrogate model. As pseudoviruses mimic the virion surface of the native virus, they are often used as a surrogate model for examining the initial steps of viral infection (e.g., viral entry). They therefore represent a good model for testing neutralizing antibodies [51] and viral inactivation protocols [52]. The SARS-CoV-2pp were generated by transfecting HEK 293FT cells with a luciferase reporter-tagged HIV-1-based lentiviral backbone along with a plasmid encoding the SARS-CoV-2 S protein. The titer was determined based on the luciferase signals yielded after Huh-7 cells infection with the serial dilution of SARS-CoV-2pp stock. As shown in Figure 1, SARS-CoV-2pp could successfully enter Huh-7 cells and produce luciferase signals, which correlate with the dilution of viral titer. The SARS-CoV-2pp stock was then used for the following experiments.

### 3.2. Elimination of S/D Cytotoxicity by C18 Filtration in S/D-Treated Plasma

**Figure 2:**
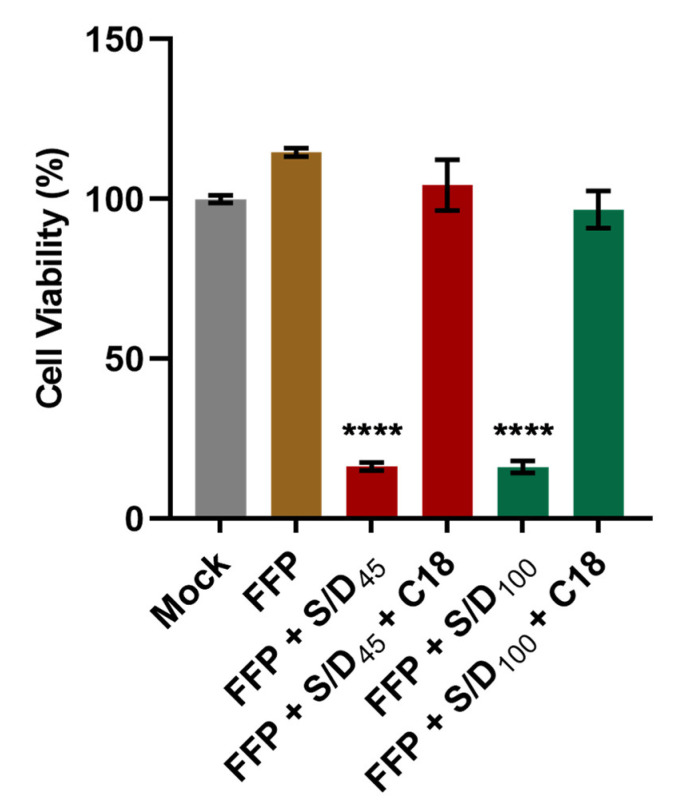
**C18 filtration efficiently removes S/D cytotoxicity.** Huh-7 cells were treated with basal medium (Mock), freshly frozen plasma (FFP), plasma treated with 1% TnBP/1% Triton X-45 (FFP + S/D_45_), plasma treated with 1% TnBP/1% Triton X-45 followed by C18 cartridge filtration (FFP + S/D_45_ + C18), plasma treated with 1% TnBP/1% Triton X-100 (FFP + S/D_100_), or plasma treated with 1% TnBP/1% Triton X-100 followed by C18 cartridge filtration (FFP + S/D_100_ + C18) for 72 h before cell viability was measured by CCK-8 assay. Data shown were collected in duplicate and expressed as mean ± standard deviation (SD). Statistical significance was analyzed by one-way ANOVA with Dunnett’s multiple comparisons test (**** *p* ≤ 0.0001 compared to Mock).

Before the infectivity experiment, we tested the elimination of the toxicity associated with both S/D combinations (S/D_45_ or S/D_100_) in plasma by using C18 reverse phase chromatography cartridges (Sep-Pak Plus C18 cartridge) to avoid any toxic effects related to the samples during the infectivity assays in cell cultures. To confirm the clearance of S/D by C18 filtration in plasma after treatment, we performed a cell viability assay using FFP treated with S/D (S/D_45_ or S/D_100_) with or without C18 filtration in Huh-7 cells. As shown in Figure 2, while significant cell death was generated with the samples of FFP treated with S/D (S/D_45_ or S/D_100_), no cytotoxicity in Huh-7 cells was observed with the samples of FFP alone and C18 filtrates of FFP treated with S/D. These results indicate that the filtration through the hydrophobic C18 could effectively eliminate the toxicity of each S/D combination from plasma after treatment.

### 3.3. S/D Inactivation Protocols Efficiently Eliminate SARS-CoV-2pp Infectivity in Human Plasma

**Figure 3:**
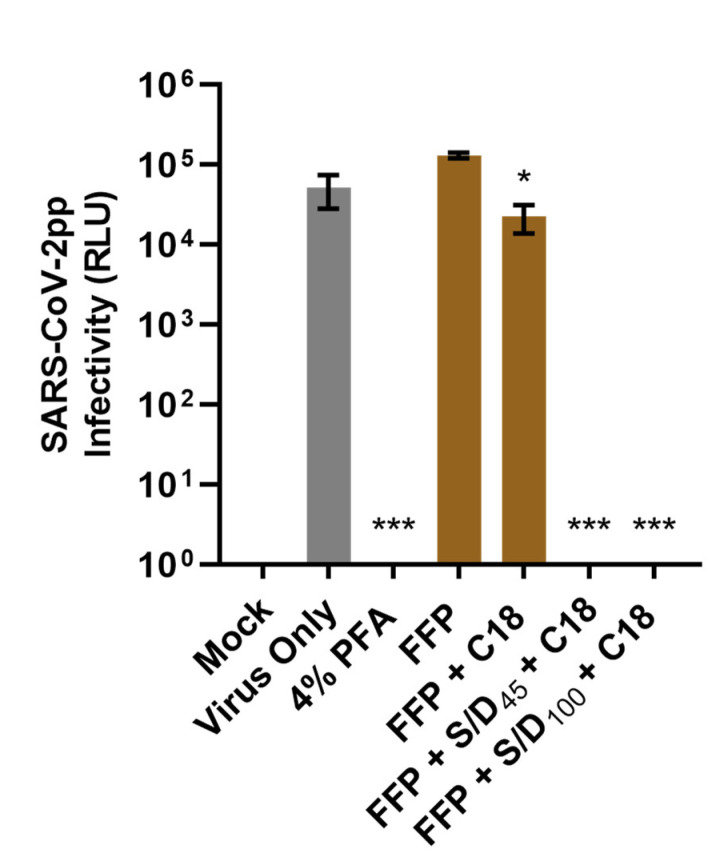
**S/D treatments completely inactivate SARS-CoV-2pp from plasma.** Huh-7 cells were infected with SARS-CoV-2pp-spiked freshly frozen plasma (FFP), SARS-CoV-2pp-spiked FFP treated with C18 cartridge filtration (FFP + C18), SARS-CoV-2pp-spiked FFP treated with 1% TnBP/1% Triton X-45 followed by C18 cartridge filtration (FFP + S/D_45_ + C18), or SARS-CoV-2pp-spiked FFP treated with 1% TnBP/1% Triton X-100 followed by C18 cartridge filtration (FFP + S/D_100_ + C18) for 1.5 h. Viral infectivity was measured by luciferase reporter activity (relative light units; RLU) after 72 h incubation. Data shown are collected in triplicate and expressed as mean ± standard deviation (SD). Statistical significance was analyzed by one-way ANOVA with Dunnett’s multiple comparisons test (* *p* ≤ 0.05 and *** *p* ≤ 0.001 compared to Virus Only).

To demonstrate whether S/D treatments can eliminate SARS-CoV-2 infectivity in human plasma, we used S/D-treated or -untreated SARS-CoV-2pp-spiked FFP to infect Huh-7 cells. Both S/D treatments followed by C18 filtration of plasmas completely eliminated the infectivity of SARS-CoV-2pp, resulting in an undetectable level of luciferase reporter signals, similar to the Mock and PFA fixation control (Figure 3). These data show the treatments of plasma with S/D (S/D_45_ or S/D_100_) followed by C18 filtration could efficiently abolish SARS-CoV-2pp infectivity (decrease over 4 log_10_). In addition, the infectivity of SARS-CoV-2pp-spiked FFP treated with C18 cartridge filtration (FFP + C18) was modestly decreased, suggesting that the C18 filtration could additionally decrease SARS-CoV-2pp in the plasma.

### 3.4. The Neutralizing Activity of COVID-19 Convalescent Serum Is Preserved Following the S/D Inactivation Protocols

**Figure 4:**
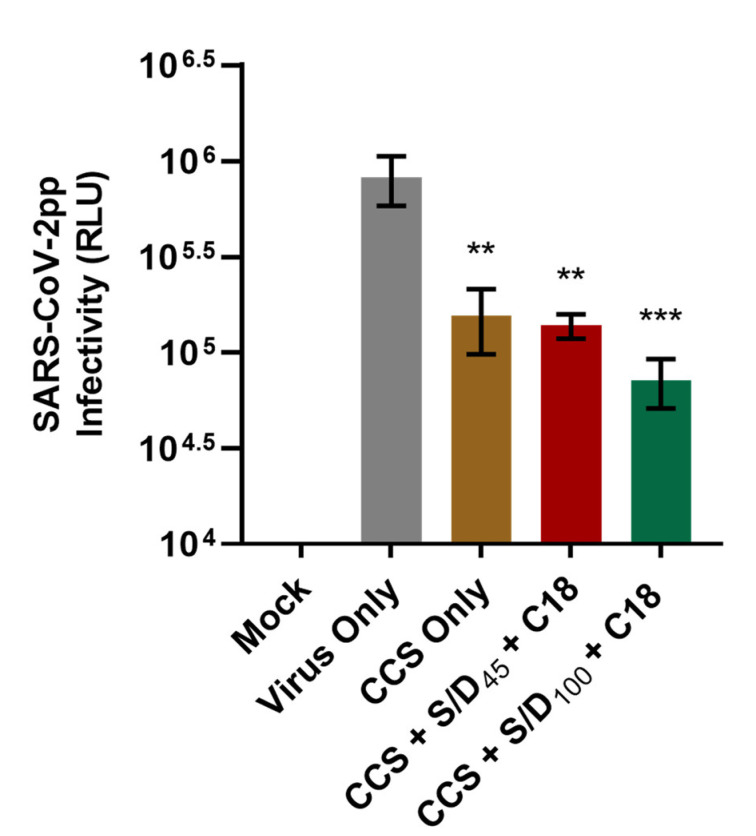
**Neutralization efficacy of SARS-CoV-2 nAbs in COVID-19 convalescent serum (CCS) were preserved after the S/D inactivation protocols.** Patient-derived CCS was treated with the S/D inactivation protocols (CCS + S/D_100_ + C18 or CCS + S/D_45_ + C18) or left untreated (CCS Only) before incubation with SARS-CoV-2pp at 37 °C for 1 h in an Eppendorf tube. The CCS–virus mixture was then used to infect Huh-7 cells, and viral infectivity was measured by luciferase reporter activity (relative light units; RLU) after 72 h incubation. Data shown were collected in triplicate and expressed as mean ± standard deviation (SD). Statistical significance was analyzed by one-way ANOVA with Tukey’s multiple comparisons test (** *p* ≤ 0.01 and *** *p* ≤ 0.001 compared to Virus Only).

To verify whether the neutralizing activity of convalescent serum could be preserved following the S/D treatment, we next treated a patient-derived CCS with the S/D inactivation protocol. As shown in Figure 4, CCS (CCS Only) significantly reduced SARS-CoV-2pp infectivity, and CCS treated with the S/D inactivation protocols (CCS + S/D_100_ + C18 and CCS + S/D_45_ + C18) could also produce the same effect. No statistically significant difference in luciferase activity was observed between the S/D-treated and -untreated CCS samples. Our results suggested that both S/D inactivation protocols preserved the neutralizing activity of the SARS-CoV-2 nAbs in the CCS.

## 4. Discussion

SARS-CoV-2 pseudoviruses are useful for virological applications such as viral entry studies, drug screening, Abs detection, and the investigation of the S protein [53]. Here, we have shown the utility of SARS-CoV-2 S-pseudotyped lentiviral particles (Figure 1) for validation of viral inactivation protocols for therapeutic blood products. The development of the pseudoviral particle neutralization test (PPNT) is effective to quantify the neutralization titers of SARS-CoV-2 nAbs in the CCP safely in typical biosafety level (BSL)-2 laboratory facilities of blood centers, which will allow them to select the CCP units for therapeutic use [41].

**Figure 5:**
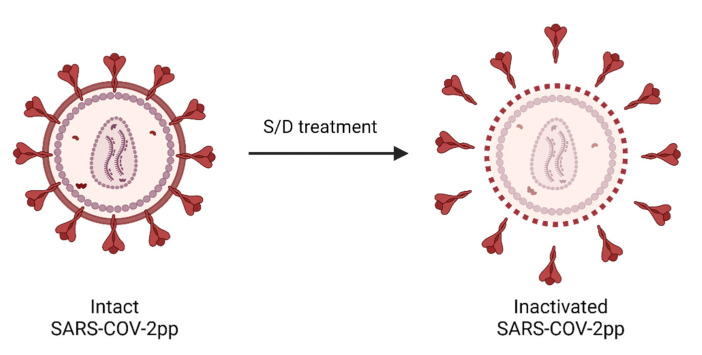
**Schematic illustration of S/D treatment’s impact on inactivating SARS-CoV-2pp.** The intact lentivirus-based SARS-CoV-2pp is inactivated after S/D treatment, which disrupts the lipid membrane of enveloped viruses, thus destabilizing particle integrity and preventing viral infection. Created with BioRender.com.

S/D treatment is a standard method used to inactivate viruses in human blood products [54]. Such a viral inactivation method can safely inactivate all lipid-enveloped viruses in plasma [44], which includes coronaviruses. The action of S/D treatment is fast, and its inactivation ability can usually be achieved within the first 15 min [55]. The mechanism consists in disrupting the lipid membrane of enveloped viruses [56]. As such, the nonionic detergents used in the S/D treatment protocol could effectively disrupt the viral membrane of SARS-CoV-2pp (Figure 5) and inactivate the particle for infection. Since the target of S/D treatment is on the lipid viral membrane, such inactivation activity should not be affected by the mutations of viral proteins in newly emerging variants, even if their binding stability to the ACE2 receptor were enhanced [57]. In addition, the S/D methods are attractive in the manufacturing process of intravenous immunoglobulins (IVIg). They appear to have no detrimental effect on the binding capacity and functions of IgG [56] with good recovery of the product during the process [58]. The S/D procedures commonly incorporated in the purification process of the immunoglobulins consist of using the combination of 0.3–1% TnBP with one, sometimes two, detergents such as Tween-80, Triton X-100, or sodium cholate. Most processes are carried out at 20–35 °C for at least 1–6 h [59]. In the past, S/D (1% TnBP/TritonX-100) treatment has been found effective in inactivating SARS-COV (≥5.75 ± 0.25 log_10_ after 30 min incubation period) in immunoglobulin preparations [60]. Two main combinations of S/D (typically 1% TnBP/1% TritonX-100 and 1% TnBP/1% TritonX-45) are also described in the treatment of plasma for transfusion. Tritons (Triton X-100 or Triton X-45) are nonionic detergents belonging to octylphenoxy polyoxyethylene ethers. They have the same hydrophobic moiety, but the hydrophilic moiety contains a different number of oxyethylene units [61]. The combination of 1% TnBP/1% Triton X-100 was the first viral inactivation method implemented on a large pool of donations on an industrial scale [62], whereas the combination of 1% TnBP/1% Triton X-45 followed by oil extraction and filtration was incorporated in a closed bag system for the treatment of a mini pool of plasma [44].

Since its implementation in the processes of preparing plasma products and plasma, S/D technology has contributed to stopping the transmission of viral infections (e.g., hepatitis B and C, HIV) by plasma products [62,63,64]. Additionally, S/D treatment is known to be effective in inactivating (re)emerging lipid-enveloped viruses that remain a challenge for blood transfusion services such as West Nile virus, dengue virus, Zika virus, and Chikungunya virus [65]. During the COVID-19 pandemic, many regulatory authorities recommended the implementation of PRT to maximize the pathogen safety of CCP [66]. Our experiment has shown that the treatments of SARS-CoV-2pp-spiked plasma with S/D combinations (S/D_45_ or S/D_100_) eliminated SARS-CoV-2pp infectivity to the undetectable level (Figure 3). We also demonstrated the C18 filtration, which is used to remove the reactants of S/D agents for industrial plasma for transfusion, could additionally reduce SARS-CoV-2pp infectivity. In a study published by Horowitz et al., they also found that C18 column could remove 1 log_10_ TCID_50_ of HIV [62].

Other PRTs using photochemical treatments, such as methylene blue/visible light (MB + visible light), amotosalen/UVA (A + UVA), and riboflavin/UVB (R + UVB), are available in blood centers for single-donor plasma donation treatments. Their mechanisms of action lead to inactivating viruses by alteration of nucleic acids [49]. It has been shown that MB + visible light reduced SARS-COV (≥3.1 log_10_) [67] and MERS-COV (≥3.3 log_10_) [68] in plasma. R + UVB treatment could reduce ≥4.0 log_10_ for MERS-COV [69] and ≥3.40 log_10_ [70] or ≥4.79 for SARS-CoV-2 [66] in plasma. Similarly, A+ UVA reduced >3.32 ± 0.2 for SARS-CoV-2 [71] and ≥4.67 ± 0.25 for MERS-COV [72] in treated plasma. Our results using the S/D treatment followed by C18 filtration represent an additional viral inactivation protocol against SARS-CoV-2.

There are currently in the literature controversial data on the immunological properties of Abs in CCP treated with various PRT available in blood centers using either viral neutralization assays or high-throughput serology. Kostin et al. found in general a significant decrease in nAbs in CCP in all three photochemical PRT. They concluded that MB + visible light or A + UVA treatments seem to preserve the immunological properties of nAbs in CCP compared to R + UVB treatment [40,66]. However, Franchini M et al. observed no statistically significant difference between preinactivation and postinactivation nAb titers during A + UVA treatment [73]. Yonemura et al. also suggested that the functionality of nAbs in CCP did not alter after R + UVB treatment [41]. Moreover, the investigation of the impact of MB + visible light treatment on the immunological properties of Abs against Ebolavirus in plasma showed the preservation of the binding ability of IgM and IgG to their epitopes or the Fc receptor interaction of IgG [74]. A slight reduction [75] or no reduction [76] of Ebolavirus nAbs were found in CP treated with A + UVA treatment. In addition to the aforementioned PRT, S/D treatments with Triton X-45 or Triton X-100 are regarded to have minimal impact on the functionality and neutralizing activity of fractionated immunoglobulins [59,77], but data on anti-SARS-CoV-2 are lacking. To clarify this, we demonstrated that 30 min of S/D treatments (S/D_45_ or S/D_100_), which is enough to inactivate SARS-CoV-2pp, does not impact the functionality of SARS-CoV-2 nAbs in CCS (Figure 4). As such, the S/D inactivation protocol presents a useful strategy for removal of SARS-CoV-2 infectivity while preserving anti-SARS-CoV-2 nAbs functionality in CCP/CCS-based blood products.

## 5. Conclusions

Our investigation demonstrated that S/D treatments with TnBP and Triton X-45 or Triton X-100 followed by C18 filtration effectively removed SARS-CoV-2pp infectivity from plasma without destroying the functionality of nAbs against SARS-CoV-2. We believe S/D treatments could be useful in viral inactivation/elimination processes of therapeutic blood products against SARS-CoV-2.

## Data Availability

Data are contained within the article.
